# Supplementary material for: Sub-kBT micro-electromechanical irreversible logic gate
Source: Nat Commun. 2016 Jun 28;7:12068. doi: 10.1038/ncomms12068 (PMC4931245; doi:10.1038/ncomms12068)
Supplement: Supplementary Information — Supplementary Figures 1-3, Supplementary Table 1, Supplementary Methods. [file ncomms12068-s1.pdf]

Supplementary Information

Supplementary Figures

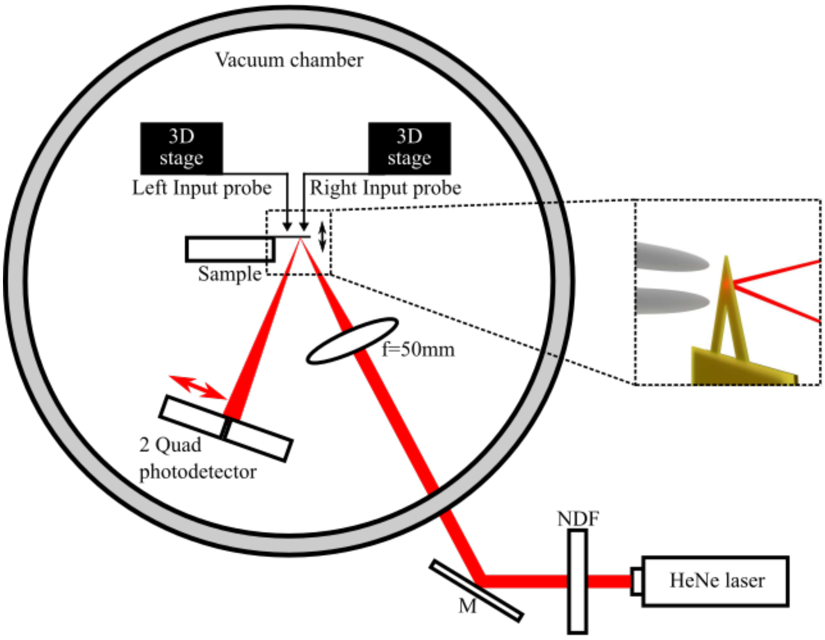

Supplementary Figure 1 - Scheme of the setup used for the measurements. The red line is a representation of the optical path of the laser used for the measurements.

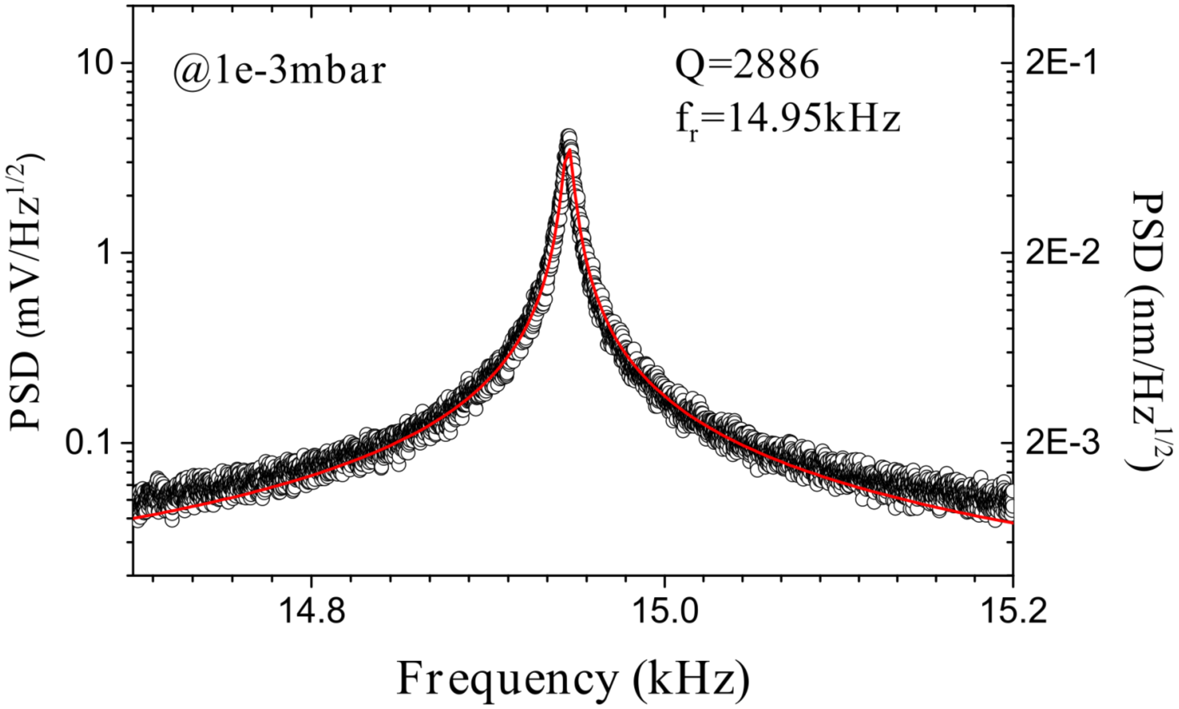

Supplementary Figure 2 - Power Spectral Density under thermal noise. Dots are experimental data

relative to the photodiode voltage output; continuous line is the Lorenzian fit.

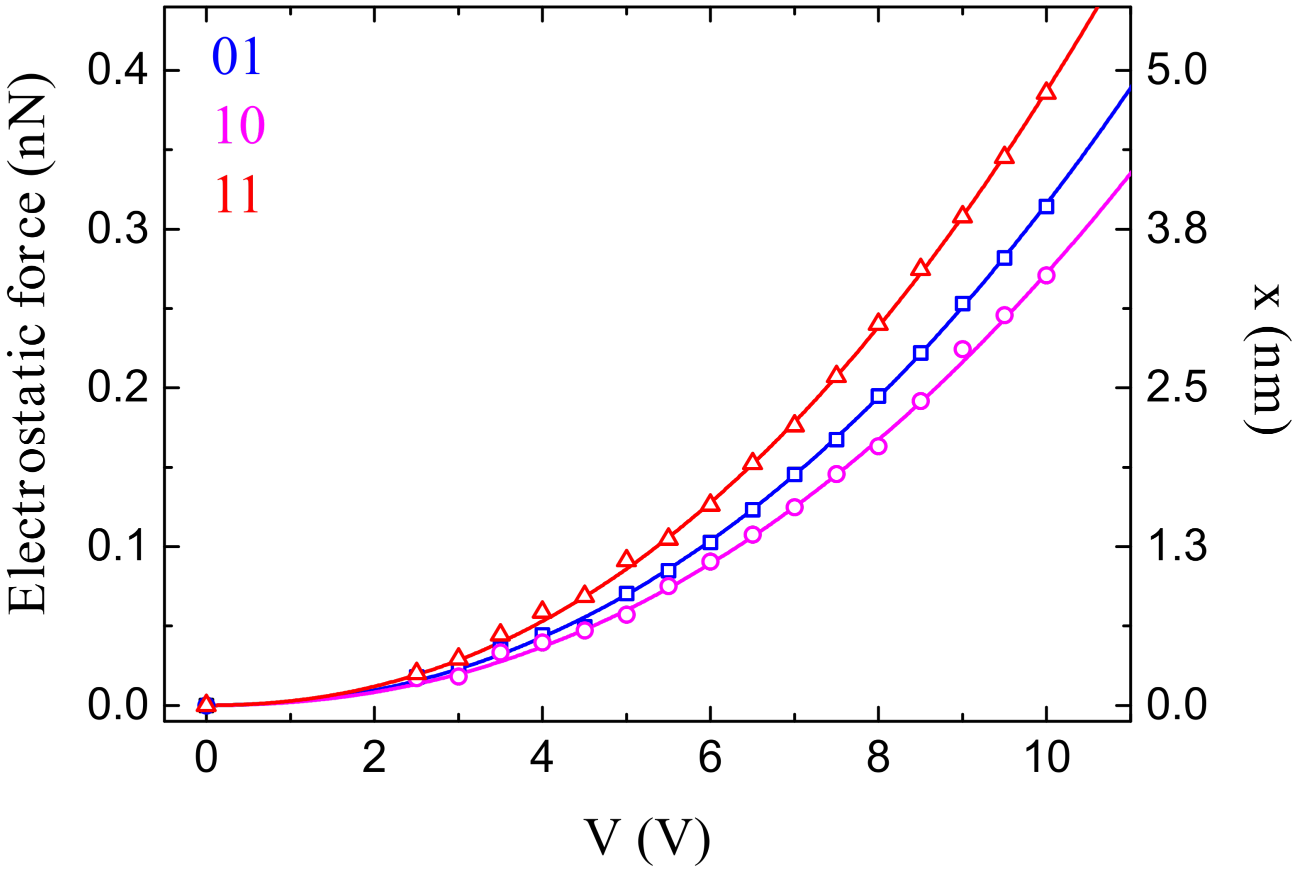

Supplementary Figure 3 - Relation between tip deflection (right axes) and voltage in the probes. The related values for the applied force on the cantilever are also shown (left axes). Points are experimental data while continuous lines represent the fit.

Supplementary Tables

	α	γ
Input 01	4.167e-23	2.183
Input 10	3.981e-23	2.184
Input 11	6.39e-23	2.174

Supplementary Table 1 - Fitted values for α and γ for the three different inputs.

Supplementary Methods

Experimental setup calibration. The deflection of the cantilever, x, is measured with an AFM-like optical lever (see Supplementary Fig. 1): a small bend of the cantilever provokes the deflection of a

laser beam incident to the cantilever tip that can be detected with a two quadrants photo detector. The laser beam is focused on the cantilever tip with an optical lens (focal length f=50 mm).

For small cantilever deflections the response of the photo detector remains linear, thus $x = r_{\mathrm{x}}\Delta V_{\mathrm{PD}}$, where ΔV_{PD} is the voltage difference generated by the two quadrants of the photo detector.

In order to determine r_{x} we look at the frequency response of the system under thermal excitation. Modeling the cantilever as a harmonic oscillator its characteristic transfer function can be written as:

$$G(\omega) = \sqrt{\frac{4k_{\mathrm{B}}T}{Qk\omega_0}}\sqrt{\frac{1}{\left(1-\frac{\omega}{\omega_0}\right)^2 + \frac{\omega^2}{Q^2{\omega_0}^2}}} \quad (1)$$

Where T is the temperature, k_{B} is the Boltzmann constant, k the elastic constant, ω_0 the angular resonant frequency and Q the quality factor. For $\omega \ll \omega_0$, $G(\omega)$ approaches the thermomechanical noise limit

$$x_{\mathrm{s}} = \sqrt{\frac{4k_{\mathrm{B}}T}{Qk\omega_0}} \quad (2)$$

The cantilever Power Spectral Density (PSD) has been measured and compared with the ideal transfer function (see Supplementary Fig. 2). The relation between the measured V_{s} and the expected x_{s} gives the value for $r_{\mathrm{x}} = 2.1256 \cdot 10^{-8}$ m V^{-1}. The PSD has been also used to estimate the quality factor, Q, and the resonant frequency of the first mode, f_{r}.

Force calibration. Electrostatic forces are applied through two electrostatic probes mounted on two 3D stages for a fine control of their position (minimum step lesser than 20 nm). With the assumption of a harmonic oscillator the restoring force can be expressed as $F = -kx$. The relation between applied voltage to the probe and the force acting on the cantilever has been estimated from the knowledge of the spring constant and the displacement x in static conditions. The calibration result is presented in Supplementary Fig. 3. The data obtained for all input combinations ("01", "10" or "11") fit the model $F = \alpha\frac{V^{\gamma}}{(g-x)^2}$ where $g = 5\ \mu m$ is the distance between the probe and the cantilever. The fitted values for α and γ for the three cases are reported in Supplementary Table 1.
